# Meiosis-Specific Stable Binding of Augmin to Acentrosomal Spindle Poles Promotes Biased Microtubule Assembly in Oocytes

**DOI:** 10.1371/journal.pgen.1003562

**Published:** 2013-06-13

**Authors:** Nathalie Colombié, A. Agata Głuszek, Ana M. Meireles, Hiroyuki Ohkura

**Affiliations:** The Wellcome Trust Centre for Cell Biology, School of Biological Sciences, The University of Edinburgh, Edinburgh, United Kingdom; Stowers Institute for Medical Research, United States of America

## Abstract

In the oocytes of many animals including humans, the meiotic spindle assembles without centrosomes. It is still unclear how multiple pathways contribute to spindle microtubule assembly, and whether they are regulated differently in mitosis and meiosis. Augmin is a γ-tubulin recruiting complex which “amplifies” spindle microtubules by generating new microtubules along existing ones in mitosis. Here we show that in *Drosophila melanogaster* oocytes Augmin is dispensable for chromatin-driven assembly of bulk spindle microtubules, but is required for full microtubule assembly near the poles. The level of Augmin accumulated at spindle poles is well correlated with the degree of chromosome congression. Fluorescence recovery after photobleaching shows that Augmin stably associates with the polar regions of the spindle in oocytes, unlike in mitotic cells where it transiently and uniformly associates with the metaphase spindle. This stable association is enhanced by **γ**-tubulin and the kinesin-14 Ncd. Therefore, we suggest that meiosis-specific regulation of Augmin compensates for the lack of centrosomes in oocytes by actively biasing sites of microtubule generation within the spindle.

## Author Summary

Although centrosomes are the main sites of microtubule assembly in mitotic cells, the meiotic spindle assembles without centrosomes in the oocytes of many animals including humans. It has also been shown that bipolar spindles can be assembled in mitotic cells even when functional centrosomes are artificially eliminated. It is still unclear how spindle microtubules assemble without centrosomes, and whether microtubule assembly is regulated differently in mitosis and meiosis. Here we investigated the role and regulation of the conserved protein complex Augmin, which recruits **γ**-tubulin to microtubules to generate new microtubules, in *Drosophila* oocytes. We found that meiosis-specific regulation of Augmin substitutes for a lack of centrosomal activity in oocytes by biasing microtubule assembly towards poles. As Augmin is conserved widely in higher eukaryotes, our finding has an important implication in our understanding of chromosome segregation and mis-segregation in human oocytes.

## Introduction

Spindles can assemble without centrosomes naturally in oocytes and artificially in mitotic cells [1]–[3]. This report asks whether the meiotic spindle is simply a mitotic spindle without centrosomes, or if oocytes have developed specific mechanisms which compensate for the absence of centrosomes. Centrosome-dependent and chromosome-dependent pathways are two well documented pathways which generate spindle microtubules [4]. Recently another pathway has been described. This pathway generates new microtubules from the side of existing spindle microtubules [5], and therefore depends on other microtubule assembly pathways. A new microtubule branches at a low angle and with the same polarity as the original filament, and is mediated by the 8-subunit γ-tubulin recruiting complex Augmin in mitosis [6], [7], [8]. Augmin subunits are functionally interdependent, and require each other for protein stability [6], [9]. The Augmin complex was originally identified in *Drosophila*, but shown to be conserved widely among higher eukaryotes [6], [7], [10]–[15].

The Augmin complex is associated uniformly with spindle microtubules, recruits γ-tubulin onto spindle microtubules, and increases both the density of spindle microtubules during mitotic metaphase as well as the density of central spindle microtubules during mitotic anaphase [6], [9], [10], [16]. Therefore, Augmin's function in mitosis is proposed to amplify microtubules by generating new microtubules along existing spindle microtubules [6]. The localisation of Augmin to centrosomes or centrosomal regions is shown in mammalian interphase and in *Drosophila* mitotic prophase, although the significance of these localisations has not been demonstrated [7], [11], [17], [18]. The microtubule binding activity of the human Augmin complex is regulated by phosphorylation by Plk1 and Aurora A [19], [20].

Mutations of Augmin subunits have been isolated in Drosophila and lead to female sterility [9], [16]. In mutant oocytes, the spindle microtubules are robustly assembled with chromosomes misaligned and homologous centromeres further apart [9]. This suggests that in oocytes Augmin plays a different role from that in mitosis. Here we report meiosis-specific regulation of this γ-tubulin recruiting complex that may function to substitute for the lack of the centrosomal activity in oocytes.

## Results

### Augmin plays a crucial role in limiting chromosome spreading within the spindle

**Figure 1:**
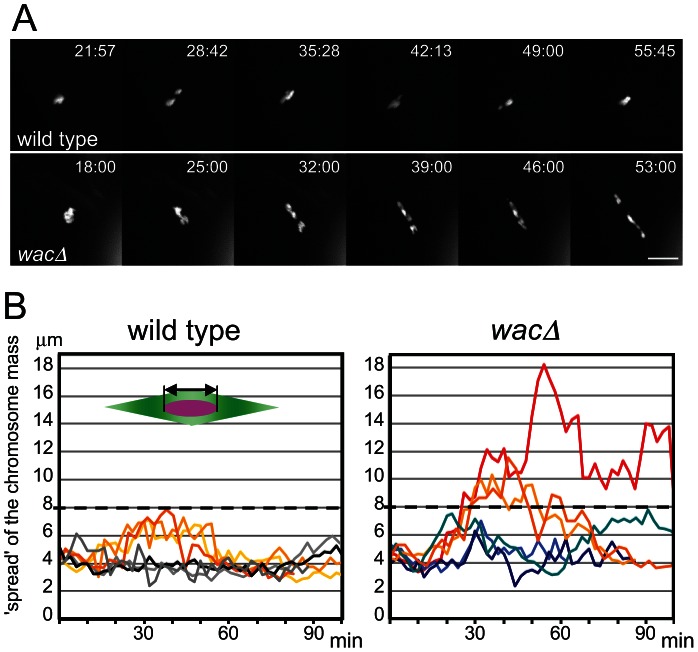
Chromosomes fail to congress in *wac* mutant oocytes. (A) Chromosome movement in wild-type and *wacΔ* oocytes expressing Rcc1-mCherry. Scale bar = 10 µm. Time = min:sec. (B) The degree of chromosome congression in wild-type and *wacΔ* oocytes. The spread of the chromosome mass along the spindle axis, excluding the 4^th^ chromosome (the double arrow in the diagram), in six oocytes each plotted from nuclear envelope breakdown (time 0) over time.

We have previously isolated a null mutant of the core Augmin subunit Wac, in which other subunits of the Augmin complex are also destabilised [9]. In the mutant oocytes, chromosomes are misaligned with homologous centromeres mostly bi-oriented, but further apart within the robustly assembled meiotic spindle. In later stages, chromosomes are mis-segregated at a very high frequency, leading to female sterility [9]. To establish the precise role of Augmin, we followed chromosome behaviour and microtubule assembly in live wild-type and mutant oocytes from nuclear envelope breakdown onwards (Figure 1A, B; Movies S1, S2). When the nuclear envelope breaks down, chromosomes were clustered together in both wild-type and mutant oocytes. Then, in wild-type oocytes, individual chromosomes moved within a limited range (up to 8 µm) along the spindle axis for about 30 minutes, before chromosomes were congressed to the equator of the spindle. In contrast, in most mutant oocytes, chromosomes tended to cover a wider range than wild type and spread widely along the spindle axis from the start of spindle formation. Chromosomes eventually congress in most, but not all, older oocytes (Figure S1). Therefore Augmin is required for limiting the range of chromosome movement to keep chromosomes at the spindle equator especially in early stages.

### Augmin is dispensable for bulk spindle microtubule assembly

**Figure 2:**
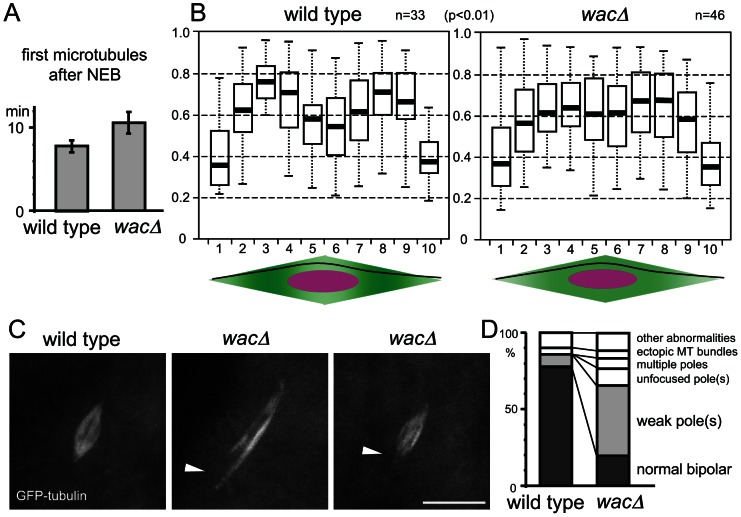
Augmin facilitates the generation of microtubules near spindle poles. (A) Timing of the first microtubule assembly from nuclear envelope breakdown in wild-type and *wacΔ* mutant oocytes. The error bars are SEM. n≥11, p = 0.06. (B) Normalised tubulin intensity plots along the long axis of the wild-type and *wacΔ* mutant spindles. Pixel intensity was measured along a line from one pole to the other as in the diagrams below. Box plots show the central 50% of the data (box), the median (central bisecting line), and 1.5X the interquartile range (whiskers). The tubulin intensity of the sub-polar spindle regions (the regions 3,8) relative to that of the equator region (5,6) is significantly lower in the *wacΔ* mutant than wild type (p<0.01). (C) Spindle poles are often missing or weak in *wacΔ* oocytes expressing GFP-tubulin, while they are robust in wild-type oocytes expressing GFP-tubulin. Scale bar = 10 µm. (D) The frequencies of various spindle morphologies in wild-type and *wacΔ* oocytes expressing GFP-tubulin. The spindles with at least one weak or missing pole were significantly more frequent in the *wac* mutant (p<0.01, n≥48).

Previous studies showed that in mitosis, Augmin assembles the majority of spindle microtubules independently of centrosomes [6], [9], [16], [17]. In contrast, our previous observation from fixed oocytes indicated that metaphase I spindles in a *wac* null mutant show no significant difference in microtubule density from those in wild type [9]. To investigate the kinetics of meiotic spindle assembly, live imaging was carried out in mutant and wild-type oocytes expressing GFP-α-tubulin. The timing of the appearance of the first spindle microtubules after nuclear envelope breakdown showed only a marginal difference between wild type and the mutant (Figure 2A; Figure S2). Taken together, these results indicate that unlike in mitosis of S2 cells, Augmin is largely dispensable for bulk spindle microtubule assembly in oocytes.

### Augmin is required for full microtubule assembly near the acentrosomal poles

In mitotic metaphase, Augmin is associated with spindle microtubules uniformly, and in cells depleted of Augmin, microtubule density is uniformly reduced. Therefore, it was originally proposed that Augmin amplifies (augments) microtubules by generating new microtubules alongside existing ones [6]. In fixed oocytes we previously showed that an Augmin subunit is concentrated at the polar regions of the meiotic spindle in oocytes [9]. We hypothesise that in oocytes, Augmin may generate microtubules within the metaphase spindle in a spatially biased manner.

To test this hypothesis, we quantified the spatial distribution of the microtubule density within the spindle in wild-type and mutant oocytes. Oocytes were fixed and immunostained with tubulin, and the signal intensity was measured along each spindle from one pole to the other. After normalisation, the tubulin intensity was plotted along the spindle length (Figure 2B). In the *wac* mutant, the tubulin intensity near the polar regions relative to the spindle equator is significantly lower than in wild type (p<0.01). This can be explained by a decrease of microtubule density near the poles or an increase at the spindle equator in the *wac* mutant. As Augmin is known to generate microtubules, we favour the first interpretation.

To further confirm this observation from fixed samples, we examined live oocytes using GFP-tubulin. We found a significant increase in the frequency of spindles with a missing or weak spindle pole in mutant oocytes (Figure 2C, D). Although these morphologies were not obvious in fixed samples either due to fixation or the absence of GFP-tagged protein, results from live oocytes further support the possibility of an underlying reduction in microtubules near the polar regions of the meiotic spindle. Therefore, our evidence suggests that Augmin is required for full assembly of microtubules near spindle poles in oocytes, rather than to simply amplify the existing microtubules as is seen in mitotic metaphase.

### The level of Augmin at spindle poles is well correlated with chromosome congression

**Figure 3:**
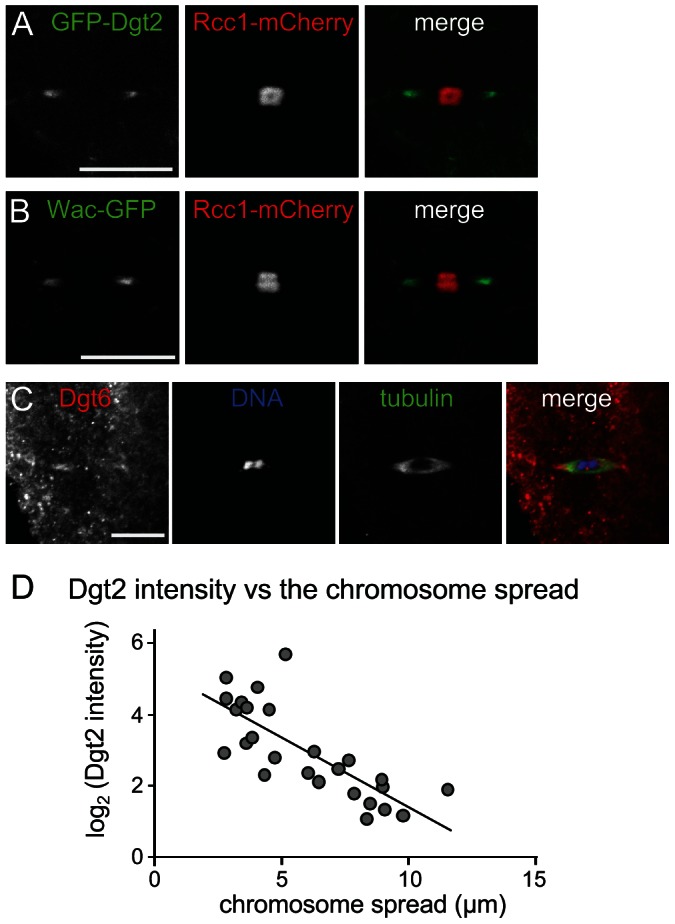
Augmin accumulation at spindle poles is correlated with chromosome congression. (A, B) GFP-Dgt2 and Wac-GFP localise to wild-type acentrosomal spindle poles. (C) Dgt6 localises to spindle poles in wild-type oocytes by immunostaining. (D) The Augmin level in spindle pole regions is well correlated with the level of chromosome congression. Live oocytes expressing GFP-Dgt2 and Rcc1-mCherry were used to measure two parameters: the spread of the chromosome mass (including the 4th chromosomes) along the spindle axis (as in Figure 1B), and the intensity of GFP-Dgt2 signal above the background (as in Methods & Materials) for each spindle. Correlation between the chromosome spread and the log of GFP-Dgt2 intensity is significant (r = −0.772, p<0.01, n = 26).

We previously showed by immunostaining that in oocytes, the Augmin subunit Dgt2 concentrates at acentrosomal spindle poles in meiotic metaphase I. This is in clear contrast to the uniform localisation of Dgt2 along spindle microtubules in mitotic metaphase. To further confirm this localisation, we generated transgenic flies which express Dgt2 tagged with GFP (GFP-Dgt2) in oocytes. Live analysis shows that GFP-Dgt2 signal was enriched in the polar regions of meiotic spindles (Figure 3A). To exclude the possibility that the Dgt2 subunit on its own, but not the Augmin complex, localises to the poles, the localisation of other Augmin subunits was examined in oocytes. Wac tagged with GFP also showed similar enrichment at the spindle poles (Figure 3B). Furthermore, immunostaining showed that Dgt6 was also concentrated to the polar regions of the spindle (Figure 3C). This behaviour of Augmin as a complex is consistent with our previous observation that the amount of Dgt2 is greatly reduced in the absence of Wac in ovaries [9]. Therefore we conclude that the Augmin complex is concentrated at the polar regions of spindles in oocytes.

We noticed that the intensity of Augmin in the polar regions of the spindle was variable from one oocyte to another. To test whether the polar accumulation of Augmin promotes chromosome congression, we measured the intensity of the GFP-Dgt2 signal at spindle poles and the degree of chromosome congression (as the length of the chromosome mass along the spindle axis) for each spindle from oocytes with various ages. We found a strong positive correlation between the intensity of Dgt2 signals in the polar region and the degree of chromosome congression (Figure 3D).

### Most Augmin complexes are stably associated with the polar regions of the spindle

In S2 cells, it has been previously shown that Augmin on the mitotic spindle turns over very rapidly (t_1/2_ of 4 seconds; [6]). To establish the molecular dynamics of Augmin on the spindle in wild-type oocytes, fluorescence recovery after photobleaching (FRAP) was used to measure the turnover rates of GFP-Dgt2 associated with the meiotic metaphase spindle in mature oocytes. After the spindle was photobleached by laser, recovery of the fluorescent signal was monitored over time. Surprisingly, the recovery was much slower in oocytes than that reported in mitotic metaphase of S2 cells.

**Figure 4:**
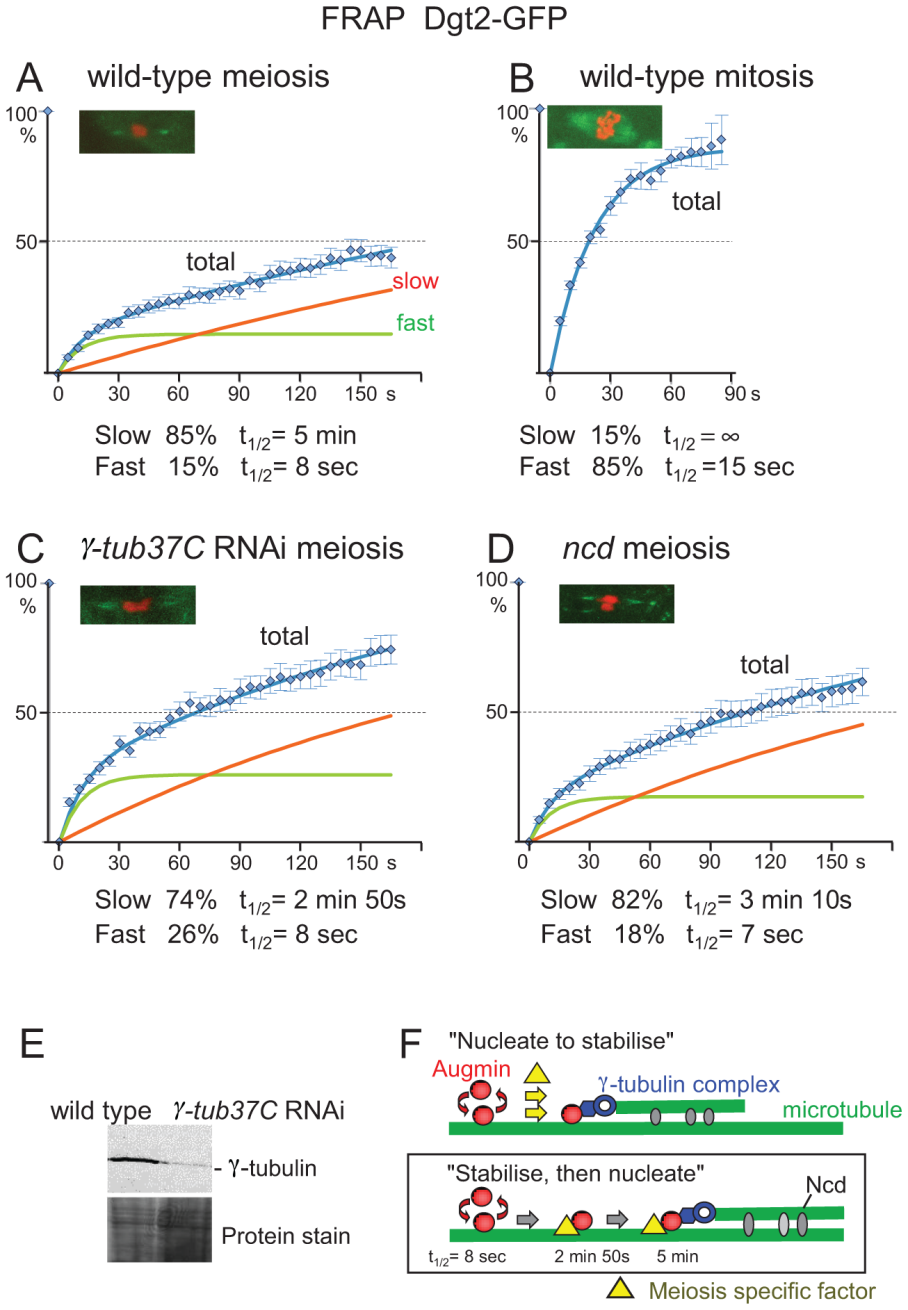
Augmin is stably associated with spindle microtubules. (A–D) FRAP of spindle-associated GFP-Dgt2 in wild-type metaphase I oocytes (A), in wild-type prometaphase/metaphase syncytial embryos (B), in oocytes depleted of γ-tubulin37C by RNAi (C), and in *ncd^D^* homozygous mutant oocytes (D). A typical meiotic figure used for FRAP is shown for each. Error bars are SEM. n≥15 in meiosis and n≥11 in mitosis. (E) Western blot of oocytes using an antibody which recognises all γ-tubulin in oocytes in wild type and after depletion of γ-tubulin37C by RNAi. (F) Two hypothetical models for stable association of Augmin with spindle microtubules. Our data are consistent with the “stabilise and then nucleate” model.

To quantify the turnover rate, recovery time of fluorescent signals were pooled together and plotted (Figure 4A). A one-population model, or a two-population model with only one turnover population did not fit to the data satisfactorily. We found that a two-population model with two distinct turnover populations fit to the observation very well. It is estimated that 85% of GFP-Dgt2 belongs to the slow population with a half turnover time (t_1/2_) of 5 minutes, and 15% belongs to the fast population with t_1/2_ of 8 seconds.

This turnover rate is much slower than an estimated value from the published data in mitotic metaphase using S2 cells [6]. However, a direct comparison is difficult due to the difference in the experimental conditions. Additionally, S2 cultured cells may not accurately represent mitotic cells in flies. Spindle defects in S2 cells depleted of Augmin appear stronger than that of neuroblast mitosis in mutants lacking Augmin which are in fact viable [6], [9], [10], [16], [17], [21], [22]. Therefore, we carried out FRAP experiments on mitotic spindles in syncytial embryos laid by the GFP-Dgt2 expressing flies (Figure 4B). The recovery curve showed that GFP-Dgt2 on metaphase mitotic spindles consists of 85% of the fast population with t_1/2_ of 15 seconds and 15% of non-turnover or very slow turnover population.

We found that Wac-GFP also showed a slow turnover in oocytes comparable to GFP-Dgt2 confirming that this is the behaviour of the Augmin complex (Figure S3). Furthermore, FRAP of GFP-tubulin indicated that microtubule dynamics is comparable between mitotic spindles in syncytial embryos and meiotic spindles in oocytes, excluding a possibility that a slow turnover of Augmin in oocytes is a mere reflection of slow microtubule turnover in oocytes (Figure S4). Therefore we conclude that the Augmin complex is associated with spindle microtubules much more stably in oocytes than in mitosis. This indicated the existence of an oocyte-specific mechanism which stabilises the association of Augmin to the polar regions of the spindle.

### γ-tubulin and Ncd contribute to the stability of Augmin on the meiotic spindle

Augmin is thought to recruit the **γ**-tubulin complex to nucleate new microtubules on existing microtubules. It is possible that the stable interaction of Augmin with the meiotic spindle is caused by its attachment to the nucleated microtubule and for some reason nucleation of microtubules is much more efficient in oocytes (“nucleate to stabilise” model; Figure 4F). In this case, a reduction of **γ**-tubulin would greatly decrease the population of stably attached Augmin.

To test this possibility, **γ**-tubulin37C, the major **γ**-tubulin in oocytes, was depleted by RNA interference (RNAi). Depletion was confirmed by Western blotting using an antibody that recognises all **γ**-tubulin isoforms (Figure 4E), and immunostaining showed a spindle defect similar to the previous report [23]. FRAP of GFP-Dgt2 showed that there is no dramatic decrease in the proportion of the slow-turnover population (85% to 74%; Figure 4C). The morphology of the spindle polar region was not significantly changed during FRAP. The most significant difference is the increase in the turnover rate of the slow population (t_1/2_ = 5 minutes to 2 minutes 50 seconds), although it is still much slower than the fast-turnover population (t_1/2_ = 8 seconds). However, as Western blot showed residual **γ**-tubulin either from incomplete depletion by RNAi or the other **γ**-tubulin isoform (**γ**-tubulin23C), the role of **γ**-tubulin may be bigger. Nevertheless, our quantitative study suggests that interaction with **γ**-tubulin is not the major cause of the stable population, but further stabilises the already stable population (“stabilise, then nucleate”; Figure 4F).

Next we looked into the involvement of Ncd (a kinesin-14), a minus-end directed motor which cross-links spindle microtubules. Ncd is not essential for viability but is important for pole focusing of acentrosomal spindles and accurate chromosome segregation in oocytes [24]. It could potentially anchor Augmin onto microtubules, or cross-link newly nucleated microtubules to existing microtubules and further stabilise the microtubule interaction of the stable Augmin population. FRAP of GFP-Dgt2 in *ncd* mutant oocytes showed that there is little change in the proportion of the slower population (85% to 82%; Figure 4D). The morphology of the spindle polar region was not significantly changed during FRAP. The most significant difference is the decrease in t_1/2_ of the slower population (5 minutes to 3 minutes 10 seconds). This suggests that interaction with Ncd does not play a major role in stabilising Augmin onto the spindle in the first place, but further stabilises the already stable population possibly by cross-linking the newly nucleated microtubule to an existing microtubule (Figure 4F).

## Discussion

**Figure 5:**
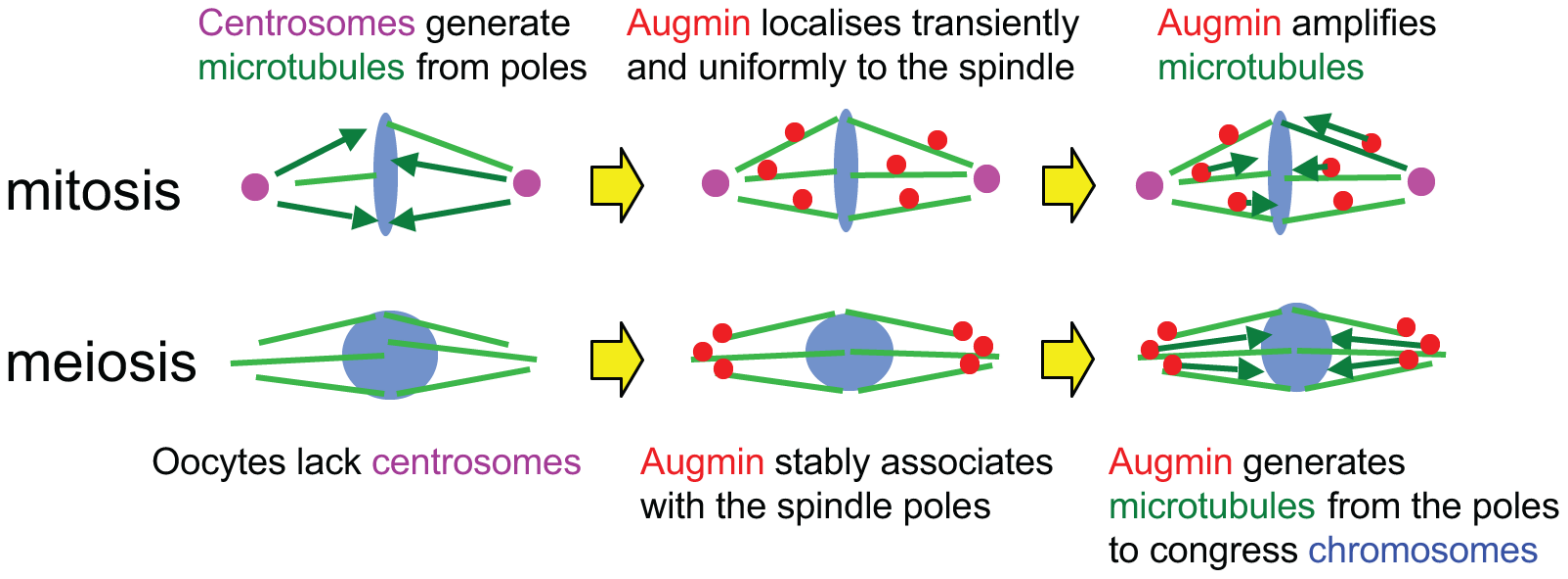
Stable association of Augmin with spindle poles compensate for the lack of centrosomes in oocytes.

Oocytes form the spindle without centrosomes in many animals including humans and *Drosophila* [1]. We propose that oocytes have specific mechanisms which compensate for the lack of centrosomal activity in meiotic metaphase (Figure 5). In mitotic prophase, Augmin concentrates to centrosomal regions [17]. In mitotic metaphase, it localises uniformly and transiently to the spindle, and generates the majority of centrosome independent microtubules (Figure 5; [6], [9], [16], [17]). In oocytes, Augmin associates with the poles of the metaphase I spindle in a stable manner, and biases microtubule assembly near the poles which lack centrosomes (Figure 5). The microtubules generated from the spindle poles may be important to congress chromosomes towards the spindle equator.

The position of chromosomes is thought to be determined by a balance of multiple forces acting on chromosomes. The main source of the forces is the kinetochore-microtubule interaction. This interaction mainly pulls, but also can push, chromosomes, and is sensitive to lack of tension [25], [26]. In addition, other forces acting on chromosome arms, often called polar ejection forces, push chromosomes to the spindle equator [25]. These forces are generated by an interaction between chromosome arms and spindle microtubules. Proposed origins of polar ejection forces include motor activities on chromosomes (chromokinesins), chromosomal proteins tracking microtubule ends and simple collision of chromosomes with polymerising microtubules [25], [27]. It is still poorly understood how chromosomes find the equator of the spindle by a balance of these forces. The organisation of spindle microtubules results in spatial differences of some forces, and chromosomes are positioned where poleward forces and anti-poleward forces are balanced. It is proposed that chromosomes are congressed at the equator as polar ejection forces are strongest near the poles and gradually decrease at the equator, which reflects the density and polarity of microtubules [25].

Polar ejection forces may play even more important roles in chromosome congression in oocytes than mitosis, but crucial differences of spindles between the two modes of divisions make it more challenging to understand. As the spindle is formed without centrosomes in oocytes [28], microtubule density is lowest near the poles [29]. This difference of the spindle geometry potentially may affect the spatial distribution of polar ejection forces and other forces. Another difference is that centromeres/chromosomes are clustered together even before the nuclear envelope breaks down [29]. After the spindle elongates from the clustered chromosomes, chromosomes initially become spread along the spindle, and then congressed. The third difference is that, without centrosomes, the position of chromosomes heavily influences the length of the spindle and the distribution of microtubules within the spindle. Furthermore, the age of the oocytes or the length of metaphase arrest increases chromosome congression and decreases spindle length [30]. We know from the observation of other mutants that more spreading of chromosomes results in longer spindles, but do not know whether the converse is correct. This intimate relationship makes it difficult to disentangle the causal relationship between spindle length/morphology and chromosome congression.

In the *wac* mutant, chromosomes are more widely spread in the meiotic spindle with homologous centromeres further apart, even relatively to the spindle length [9]. This chromosome spreading is particularly prominent in the *wac* mutant at the early stages of spindle formation, and becomes less prominent at the later stages, suggesting that an Augmin-independent polar ejection force gradually takes over though not completely. The failure or delay of proper chromosome congression in the *wac* mutant is better explained by a decrease in the pushing force acting on chromosome arms (polar ejection force) rather than an increase in the pulling force acting on kinetochores. In *wac* meiotic spindles, we found that the density of microtubules in sub-polar regions is relatively reduced, consistent with the polar concentration of Augmin in wild-type spindles. These microtubules generated near poles may be important for polar ejection forces by interacting with chromosomes directly, or through motors or microtubule end tracking proteins. We found a strong correlation between Augmin accumulation in the polar regions and chromosome congression, although the correlation does not necessarily imply a causal relationship. These findings suggest that polar microtubules generated by Augmin at the spindle poles are important for the polar ejection force which congresses chromosomes in oocytes. However, we cannot exclude alternative possibilities, such as that a *wac* mutation more indirectly affects chromosome positioning by altering the general organisation of the meiotic spindle, or the localisation of factors that influence chromosome movement or architecture. We examined thread-like projections thought to be connecting homologous heterochromatin using a phospho-H3 antibody [23], [31], but no noticeable differences were observed. Furthermore, although we did not see Augmin at the central spindle region higher than cytoplasmic background during spindle formation, we cannot exclude a possibility that Augmin may play a role there.

As we previously showed [9], *wac* mutant oocytes exhibit an elevated frequency of mono-orientation of homologous centromeres (∼10% of X chromosomes), and further show a very high incidence of chromosome mis-segregation in both stages of meiosis (∼80% and ∼70% of meiosis I and II, respectively). Similarly, in *nod* mutant oocytes which show reduced congression of achiasmatic chromosomes, it was genetically estimated that about 50% of achiasmatic X chromosomes and about 2% of chiasmatic ones mis-segregate [32]. This evidence suggests that chromosome congression or a force behind chromosome congression is crucial for accurate chromosome segregation in oocytes. Additionally, a *nod* mutant frequently shows detachment of achiasmatic chromosomes from the spindle [29], while such detachment is seen less often in the *wac* mutant. Thus Nod may play a role in anchoring chromosomes to microtubules, as well as generating a polar ejection force.

The Augmin complex is much more stably associated with spindle microtubules in oocytes than in mitotic cells. This difference is not simply explained by a difference in spindle dynamics, as the turnover of spindle microtubules is fast in both oocytes and syncytial mitosis. Interestingly, the turnover of Augmin on spindle microtubules in oocytes is significantly slower than the turnover of spindle microtubules itself. At first glance, this seems contradictory or puzzling. However, Augmin freed from one depolymerised microtubule can easily be re-captured by other microtubules in proximity rather than diffusing into the cytoplasm. Therefore the Augmin turnover between spindle and cytoplasm can be much slower than the turnover of individual microtubules. Alternatively, as our data showed a small proportion of microtubules are less dynamic, Augmin somehow could preferentially bind to these stable microtubules.

We also found that γ-tubulin and Ncd further stabilise the slow-turnover population of Augmin. One explanation is that Augmin already anchored to a microtubule is further stabilised by combined actions of γ-tubulin which nucleates a new microtubule and Ncd which crosslinks this new microtubule to existing microtubules. However, we could not exclude possibilities that these indirectly affect the turnover of Augmin by altering the spindle dynamics or organisation.

In *Xenopus* egg extract, bipolar spindles can be formed in the presence and absence of centrosomes [33]. It has also been shown that a bipolar spindle can be assembled without centrosomes in mitotic cells [2], [3]. Therefore, it is often assumed that the only difference between a mitotic and a meiotic spindle is the presence of centrosomes. But our study clearly demonstrated that a meiotic spindle in oocytes is more than a mitotic spindle without centrosomes, and meiosis-specific regulation of Augmin is a crucial part of this difference. The Augmin complex was originally identified in *Drosophila*, but shown to be conserved widely among higher eukaryotes [6], [7], [11], [12], [13], [14], [15]. Therefore our finding of meiosis-specific Augmin regulation has an important implication in our understanding of chromosome segregation and mis-segregation in human oocytes.

## Materials and Methods

### Molecular techniques

Standard DNA manipulation and immunological techniques were used throughout [34], [35]. Full-length Rcc1 was fused to mCherry and cloned into pUASp expression vector. A mutation ( AAA**ATG** to TAA**ATG** ) was introduced upstream of the ATG to reduce the expression level. Dgt2 and Wac were cloned into the Gateway expression vectors pPGW and pPWG respectively. The plasmids were injected into *w^1118^* embryos by Genetic Research Inc. For western blot, **γ**-tubulin (GTU-88; Sigma) primary antibody was used (1/500). Fluorescently labelled secondary antibodies were detected by Odyssey (Licor).

### Fly techniques

Standard techniques of fly manipulation were followed [36]. All stocks were grown at 25°C in standard cornmeal media. *w^1118^* was used as wild type. Heterozygous flies for *P[TRiP.HMS00517]attP2* and *nanos-Gal4* maternal driver were used for ***γ*** *-tubulin37C* RNAi. Details of mutations and chromosome aberrations can be found in [37] or at FlyBase (http://flybase.org) [38].

### Cytological techniques

Immunostaining of non-activated oocytes was carried out as previously described [39]. Antibodies against α-tubulin (DM1A; Sigma) and Dgt6 (1/50; [17]) were used. Live-imaging of meiosis I spindle was carried out as described [40]. Adult flies expressing Rcc1-mCherry (except for FRAP) were matured 3 to 5 days at about 21°C before dissection. Series of z-sections covering the entire spindle (separated by 1 µm) were taken every 1 to 2.5 minutes. The following transgenes were used as heterozygotes: *UASp-GFP-α-tubulin*, *GAL4* under the maternal *nanos* promotor, and *UASp-Rcc1-mCherry* on the third chromosome, and *UASp-GFP-Dgt2* and *UASp-Wac-GFP* on the second chromosome.

### Cytological analysis

The images were taken with a laser scanning confocal microscope for immunostaining and FRAP experiments, and with a spinning disc confocal for other live samples, as previously described [8], [30]. Z sections were separated by 1 µm. Images were presented as a maximum intensity projection of the z-stacks, were processed using Photoshop/ImageReady (Adobe), imageJ or Volocity, and the brightness and contrast were uniformly adjusted for the whole field without changing features of the images. Measurement of tubulin intensity throughout fixed spindles (Figure 2B) was carried out on the maximum intensity projection. Pixel intensity was measured along a line from one pole to the other. The position along the line was normalised to the spindle length. The fluorescence intensity was normalised to the maximum intensity. The spindle length was divided into 10 and the medians of pixel intensities for each division in each spindle were box-plotted. The signal intensity of sub-polar regions (divisions 3,8) relative to the equator (5,6) was significantly different between wild type and the mutant (p<0.01). *p*-values were calculated using the Wilcoxon test (Figure 2B, 3D), the Student's *t*-test (Figure 2A) and Chi square test (Figure 2C). To compare Dgt2 accumulation at the poles with the spread of chromosomes, the sum of intensity (I) was measured on maximum intensity projection in two regions of interest (ROI). The first ROI (I1, N1 = 100,000 to 160,000 pixels) includes both spindle and cytosol and the second region (I2, N2 = 500 to 3000 pixels) is the sum of the two spindle poles (including as little cytosol as possible). The following equation was then used to substract the background intensity (cytosol intensity): intensity per pixel at the poles = I2-(I1-I2)/(N1-N2). For the FRAP in wild type, ***γ*** *-tubulin37C* RNAi and *ncd^D^* mutant oocytes, the females were matured at 25°C for five days before dissection. For all the oocytes, an identical region of interest (ROI = 300×125 pixels; the pixel size is 0.1 µm) covering the whole spindle was bleached. Pictures of one pole (3z sections 1 µm apart) were taken every 5 seconds for 165 seconds. Three images were taken before bleaching. The analysis was performed on the maximum intensity projection. The fluorescence intensity was measured in an ROI of 10 pixels diameter within the pole. The average intensity for each oocyte (F(t)) was corrected for the acquisition-induced photobleaching using the average intensity (F_Control_(t)) of non-FRAPed spindles using equation 1. The corrected intensity (F_Corr_(t)) was then normalised (F_Norm_(t)) between the pre-bleach and the post-bleach intensities (t = 0) using equation 2. A model of two recovering populations (equation 3) fits well to the observations.
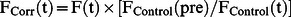
(1)

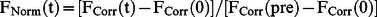
(2)

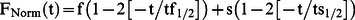
(3)where f and s are the proportion of the fast and slow populations, and tf_1/2_ and ts_1/2_ are the half-recovery times of the two populations.

For the FRAP in wild-type embryos, embryos were collected after 1.5 hour aging, manually dechorionnated and covered with halocarbon oil. For GFP-Dgt2 FRAP, an ROI (210×125 pixels) covering only one half spindle was bleached as in oocytes; for both GFP-tubulin and Wac-GFP FRAP, the ROI (210×125) covered the whole spindle. The analysis was performed as for oocytes except that the reduction of fluorescence in a non-FRAPed spindle in the same embryo was used to correct for acquisition-induced photobleaching of corresponding FRAPed spindles (the average of ≤25% after 80 s).

## Supporting Information

Figure S1 **The spread of chromosomes in relation to the age of oocytes.** For each live oocyte expressing Rcc1-mCherry, the spread of the chromosome mass along the spindle axis was measured and the stage of the oocyte was roughly estimated by the morphology of the dorsal appendages. n≥15. The frequencies of oocytes with the chromosome mass more than 10 µm and between 8 and 10 µm are indicated by bars with a dark and light shade.
(EPS)

Figure S2 **Dynamics of chromosomes and the spindle in wild-type and** *wac* **oocytes.** Time lapse images of live wild-type and *wacΔ* oocytes expressing GFP-tubulin (green) and Rcc1-mCherry (red). Time from nuclear envelope breakdown (NEB) is indicated as min:sec. Scale bar = 10 µm.
(EPS)

Figure S3 **FRAP analysis of Wac-GFP on the spindle in wild-type oocytes and syncytial embryos.** FRAP of spindle-associated Wac-GFP in wild-type metaphase I oocytes (A) and in wild-type prometaphase/metaphase syncytial embryos (B). Wac-GFP turnover is much slower in oocytes than syncytial mitosis. A model to assume two populations with different turnover rates fits well to the observation in oocytes, while a model to assume one turnover and one non-turnover population fits well to the observation in syncytial mitosis. Error bars are SEM. n = 17 in meiosis, and n = 15 in mitosis.
(EPS)

Figure S4 **Tubulin turnover on the spindle in wild-type oocytes and syncytial embryos.** FRAP of spindle-associated GFP-tubulin in wild-type metaphase I oocytes (A) and in wild-type prometaphase/metaphase syncytial embryos (B). A model to assume one turnover and one non-turnover population fits well to the both observations. More than 100% recovery in mitosis may reflect a slight increase in spindle microtubules during mitotic progression. As tubulin turnover is fast in both, it alone is unlikely to explain the difference in Augmin turnover on the spindle between mitosis and meiosis. Error bars are SEM. n = 16 in meiosis, and n = 13 in mitosis.
(EPS)

Movie S1 **Chromosome behaviour in a wild-type oocyte expressing Rcc1-mCherry. The total length of the movie is 100 minutes. The width of the frame is 30 µm.**
(MOV)

Movie S2 **Chromosome behaviour in a** *wacΔ* **oocyte expressing Rcc1-mCherry. The total length of the movie is 100 minutes. The width of the frame is 30 µm.**
(MOV)
